# Effects of PCSK9 inhibition on glucose metabolism and β-cell function in humans: a pilot study

**DOI:** 10.3389/fendo.2023.1124116

**Published:** 2023-05-31

**Authors:** Simona Moffa, Teresa Mezza, Pietro Manuel Ferraro, Gianfranco Di Giuseppe, Chiara M. A. Cefalo, Francesca Cinti, Flavia Impronta, Umberto Capece, Gea Ciccarelli, Andrea Mari, Alfredo Pontecorvi, Andrea Giaccari

**Affiliations:** ^1^ Centro Malattie Endocrine e Metaboliche, Fondazione Policlinico Universitario Agostino Gemelli IRCCS, Rome, Italy; ^2^ Pancreas Unit – Digestive Disease Center, Fondazione Policlinico Universitario A. Gemelli IRCCS, Università Cattolica del Sacro Cuore, Rome, Italy; ^3^ Unità Operativa Semplice Terapia Conservativa della Malattia Renale Cronica, Fondazione Policlinico Universitario A. Gemelli IRCCS, Rome, Italy; ^4^ Institute of Neuroscience, National Research Council, Padova, Italy

**Keywords:** PCSK 9 inhibition, diabetes, β-cell, statins, precision medicine, BMI, Dyslipidaemia

## Abstract

**Background:**

Anti-PCSK9 monoclonal antibodies are effective in reducing LDL-C and cardiovascular events by neutralizing circulating PCSK9. PCSK9, however, is also expressed in tissues, including the pancreas, and studies on PCSK9 KO mice have shown impaired insulin secretion. Statin treatment is already known to affect insulin secretion. Our aim was to conduct a pilot study to evaluate the effect of anti-PCSK9 mAb on glucose metabolism and β-cell function in humans.

**Methods:**

Fifteen non-diabetic subjects, candidates for anti-PCSK9 mAb therapy, were enrolled. All underwent OGTT at baseline and after 6 months of therapy. During OGTT, insulin secretion parameters were derived from C-peptide by deconvolution (β cell glucose sensitivity). Surrogate insulin sensitivity indices were also obtained from OGTT (Matsuda).

**Results:**

Glucose levels during OGTT were unchanged after 6 months of anti-PCSK9 mAb treatment, as well as insulin and C-peptide levels. The Matsuda index remained unchanged, while β-cell glucose sensitivity improved post-therapy (before: 85.3 ± 65.4; after: 118.6 ± 70.9 pmol min^-1^m^-2^mM^-1^; p<0.05). Using linear regression, we found a significant correlation between βCGS changes and BMI (p=0.004). Thus, we compared subjects with values above and below the median (27.6 kg/m^2^) and found that those with higher BMI had a greater increase in βCGS after therapy (before: 85.37 ± 24.73; after: 118.62 ± 26.83 pmol min^-1^m^-2^mM^-1^; p=0.007). There was also a significant correlation between βCGS change and Matsuda index through linear regression (p=0.04), so we analyzed subjects who had values above and below the median (3.8). This subgroup analysis showed a slight though not significant improvement in βCGS in more insulin resistant patients, (before: 131.4 ± 69.8; after: 170.8 ± 92.7 pmol min^-1^m^-2^mM^-1^; p=0.066).

**Conclusions:**

Our pilot study demonstrates that six-month treatment with anti-PCSK9 mAb improves β-cell function, and does not alter glucose tolerance. This improvement is more evident in patients with greater insulin-resistance (low Matsuda) and higher BMI.

## Introduction

A number of trials have highlighted an association between treatment with statins and the risk of developing type 2 diabetes (T2D), especially in people with prediabetes and/or other risk factors (e.g., family history) (1). This association with statin therapy, however, does not preclude its prescription, given the great advantage that statins have in terms of reducing cardiovascular morbidity and mortality, especially in subjects with moderate to high cardiovascular risk.

While treatment with statins still represents the most important therapeutical approach for the treatment of hypercholesterolemia, more recently, PCSK9 (proprotein convertase subtilisin/kexin Type 9) inhibitors, are being prescribed in “high risk” and “very high risk” patients, due to their effectiveness in reducing LDL-C (low density lipoprotein cholesterol) by over 60% (2, 3).

PCSK9 inhibitors are monoclonal antibodies (mAb) effective in reducing LDL-C and cardiovascular events by neutralizing circulating PCSK9. PCSK9 are mainly synthesized and secreted by the liver, but also expressed in other organs, including the endocrine pancreas.

Several studies have shown a possible role of cholesterol accumulation in inducing β-cell dysfunction (4, 5). A recent study in mice has shown that expression of LDL receptors (LDLR), and therefore the transport of cholesterol to β-cells, is also modulated by PCSK9 (6). Although randomized controlled trials investigating the safety and efficacy of evolocumab (7) and alirocumab (8) do not indicate an increased incidence of diabetes compared to the standard therapy group, a recent meta-analysis has described a modest but significant increase in glycemia and glycated hemoglobin in patients treated with anti-PCSK9 mAb, with no increase in the incidence of diabetes (9).

Studies in mouse models have shown that PCSK9 knock-out mice have a significant reduction in circulating cholesterol levels, while cholesterol content of β-cells is increased as a result of a greater expression of LDLR. These disarrangements result in the development of impaired glucose tolerance, caused by impaired insulin secretion (10). Interestingly, in the context of pancreatic islets, PCSK9 is predominantly expressed in delta cells, and it seems that this local PCSK9, rather than the hepatic-circulating one, is more involved in regulating LDLR expression in the islets (11) since mice in which PCSK9 is silenced in the liver only (mimicking the action of anti-PCSK9 mAb), have reduced circulating cholesterol levels but normal glucose metabolism (10). The same authors also demonstrated that pancreas-specific PCSK9 null mice exhibited normal blood PCSK9 and cholesterol levels but were glucose intolerant and had defective insulin secretion *in vivo*, while analysis of PCSK9-deficient islets revealed comparable β-cell mass and insulin content but impaired stimulated secretion (12). Recent reviews have evaluated the biology of PCSK9, and the effects of its functional loss in mouse models, in human carriers of LDL-lowering gene variants and in PCSK9 inhibitor-treated patients.

Collectively, the current molecular and metabolic evidence supports the safety of PCSK9 inhibitor therapy. However, with prolonged use, future clinical trials and additional meta-analyses may identify yet undiscovered patients at risk for diabetes (13–15).

So far, epidemiological studies have only evidenced the presence or absence of diabetes, which, however, also depends on other risk factors (family history, obesity and age). Anti-PCSK9 mAb could therefore modify glucose metabolism (through changes of insulin secretion) without necessarily causing diabetes. Considering that the added value of an accurate pathophysiological study is its ability to reveal the possible presence of preclinical alterations, our study aims to accurately analyze glucose metabolism and β-cell function in humans, before and after therapy with anti-PCSK9 mAb, in order to reveal potential risks of inducing T2D. In particular, the primary endpoint of the study was to evaluate the effect of six-month anti-PCSK9 mAb therapy on β-cell glucose sensitivity.

## Materials and methods

We conducted an observational pilot study, enrolling patients who were starting therapy with anti-PCSK9 mAb to evaluate the effect of this treatment on blood glucose, insulin and C-peptide levels, surrogate indices of insulin sensitivity (Matsuda) and β-cell glucose sensitivity, a parameter of β-cell function obtained from the C-peptide assay (16).

We enrolled 15 patients, all over the age of 18, (8 females and 7 males, mean age 57 ± 11) who were candidates for therapy with anti-PCSK9 mAb according to the criteria for reimbursability of the Italian national health system, at our Center for Metabolic and Endocrine Diseases at the A. Gemelli University Hospital in Rome, Italy, (see **Table 1**). The study protocol was approved by the local ethics committee (Prot. 46923/18), and all participants provided written informed consent, which was followed by a comprehensive medical evaluation.

**Table 1:** Characteristics of study population.

Subject characteristics	Before therapy	After therapy	P value
Age (years)	57 ± 11	–	–
Sex	8F/7M	–	–
BMI (kg/m^2^)	28.2 ± 5.6	28.1 ± 5.5	NS
PCSK9i	7 evolocumab/8 alirocumab	–	–
Statins	9yes/6no	–	–
Ezetimibe	15yes	–	–
LDL-C (mg/dl)	201.4 ± 53	83.1 ± 33	<0.05
FGP (mg/dl)	91.6 ± 10	91.3 ± 7.9	NS
2h-PPG (mg/dl)	118.4 ± 6.8	116.5 ± 7.6	NS
HbA1c (%)	5.6 ± 0.06	5.3 ± 0.1	NS
AUC glucose (mg/dl x min)	17908 ± 2913	16837 ± 2768	NS
AUC insulin (mcUI/ml x min)	12053 ± 7153	9865 ± 3917	NS

**Table 1** summarizes the main clinical features of study population (age, sex BMI), their basal hypolipidemic therapy and changes of LDL-C, FPG, 2h-PPG (2 hour Post Prandial Glucose) and HbA1c after treatment period. NS, Not Significant.

Inclusion criteria were: candidates for therapy with anti-PCSK9 mAb according to the Italian national health system reimbursability criteria, i.e., patients already suffering from cardio-vascular or cerebro-vascular diseases who did not reach target LDL-C despite maximal lipid lowering therapy or who were statin intolerant; subjects with familial hypercholesterolemia who did not reach target LDL-C despite maximal lipid lowering therapy or who were intolerant to statins; no known history of diabetes.

At baseline, participants underwent a standard 75 g oral glucose tolerance test (OGTT) after a 12 hour overnight fast, with measurement of glucose, insulin and C-peptide at 0, 30, 60, 90, and 120 minutes after the glucose load. We determined insulin levels using a commercial RIA kit (Medical System, Immulite DPC, Los Angeles, CA). Plasma glucose concentrations were determined by the glucose oxidase technique, using a glucose analyzer (Beckman Instruments, Palo Alto, CA, USA). Plasma C-peptide was measured by autoDELPHIA automatic fluoroimmunoassay (Wallac, Turku, Finland), with a detection limit of 17 pmol/L. OGTT glucose and HbA1c testing were used to exclude diabetes, according to the American Diabetes Association criteria (17). All samples were analyzed at our central laboratory. After six months of therapy with anti-PCSK9 mAb, during which the basal lipid lowering therapy remained unchanged, the OGTT was repeated in all subjects.

### Calculations

Insulin secretion during the OGTT was determined by C-peptide deconvolution. Beta cell function was assessed by modelling (18), based on the model-determined glucose sensitivity (βCGS), i.e., the slope of the relationship between insulin secretion and glucose concentration. C-peptide modelling was also used to calculate other secretion parameters: basal insulin secretion rate, total insulin secretion rate and rate sensitivity (19–22).

The Matsuda index (23) was calculated as index of whole-body insulin sensitivity based on insulin and glucose values obtained from the OGTT. The calculation requires glucose and insulin concentrations from a 75 g OGTT at 0, 1.5, and 2 h (2 h OGTT), including six constants optimized to match the clamp results.

### Statistics

All data are expressed as mean ± SE, unless otherwise indicated. Since samples were normally distributed, differences in means were tested by 2-tailed Student’s t test.

The correlation between βCGS and other metabolic parameters was calculated by linear mixed models for the estimation of within-person changes using the “fe” option of the xtreg command in Stata. We performed the analysis using Stata version 15.1 (StataCorp, College Station, TX). P values of 0.05 were considered statistically significant.

## Results

**Figure 1:**
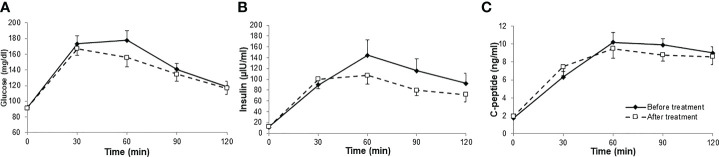
Glucose **(A)**, insulin **(B)** and C-peptide **(C)** levels during OGTT, before and after six months therapy with anti-PCSK9 mAb. N= 15 before and after treatment.

Glucose levels at OGTT remained unchanged after six months of therapy with anti-PCSK9 mAb, as did insulin and C-peptide levels (**Figure 1**).

However, we calculated AUC of both glucose and insulin curves and there were no significant differences after treatment period, as shown in **Table 1**.

**Figure 2:**
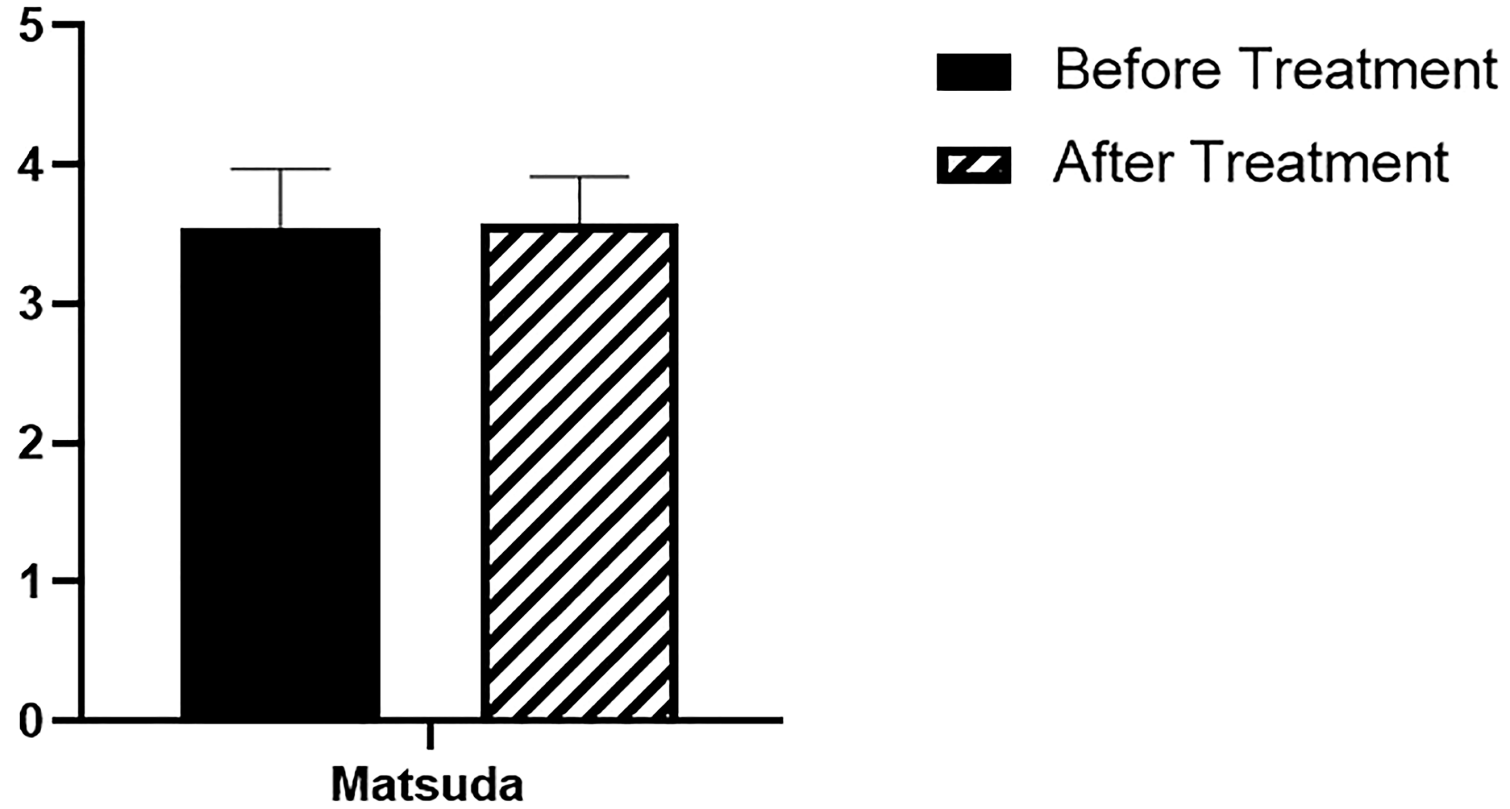
Matsuda Index. N= 15 before and after treatment.

As to the effect of six-month therapy with anti-PCSK9 mAb on surrogate indices of insulin sensitivity, we observed no significant changes in the Matsuda Index (before: 3.5 ± 1.6; after: 3.5 ± 1.3; p=NS) (**Figure 2**).

**Figure 3:**
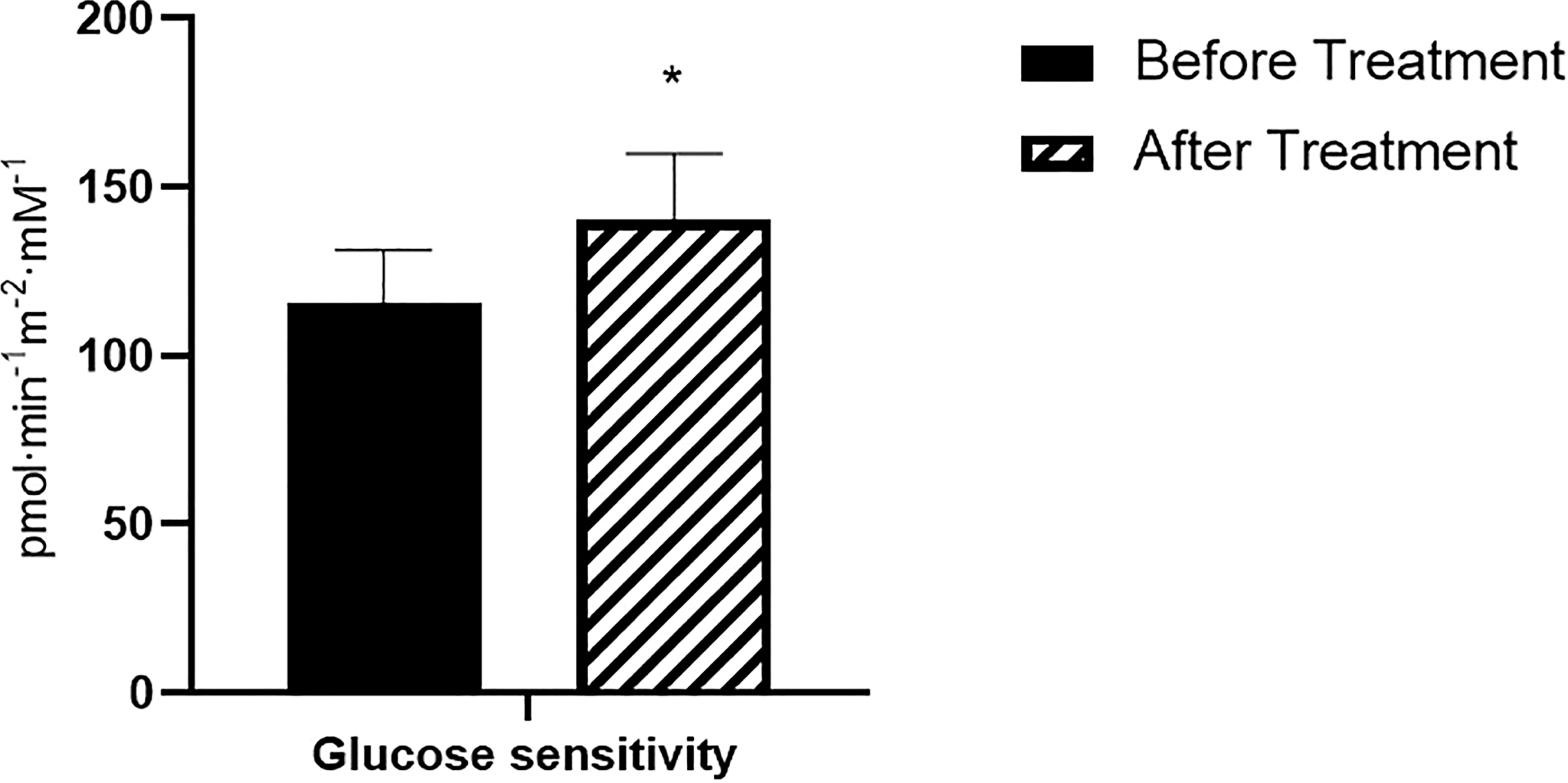
β-cell glucose sensitivity. N= 15, *P<0.05 before vs after treatment.

All insulin secretion parameters also remained unchanged after the treatment period, except for β-cell glucose sensitivity. As can be observed in **Figure 3**, there was a small but significant improvement in βCGS after the six months of anti-PCSK9 mAb treatment (before: 85.3 ± 65.4; after: 118.6 ± 70.9 pmol min^-1^m^-2^mM^-1^; p<0.05) (**Figure 3**).

**Figure 4:**
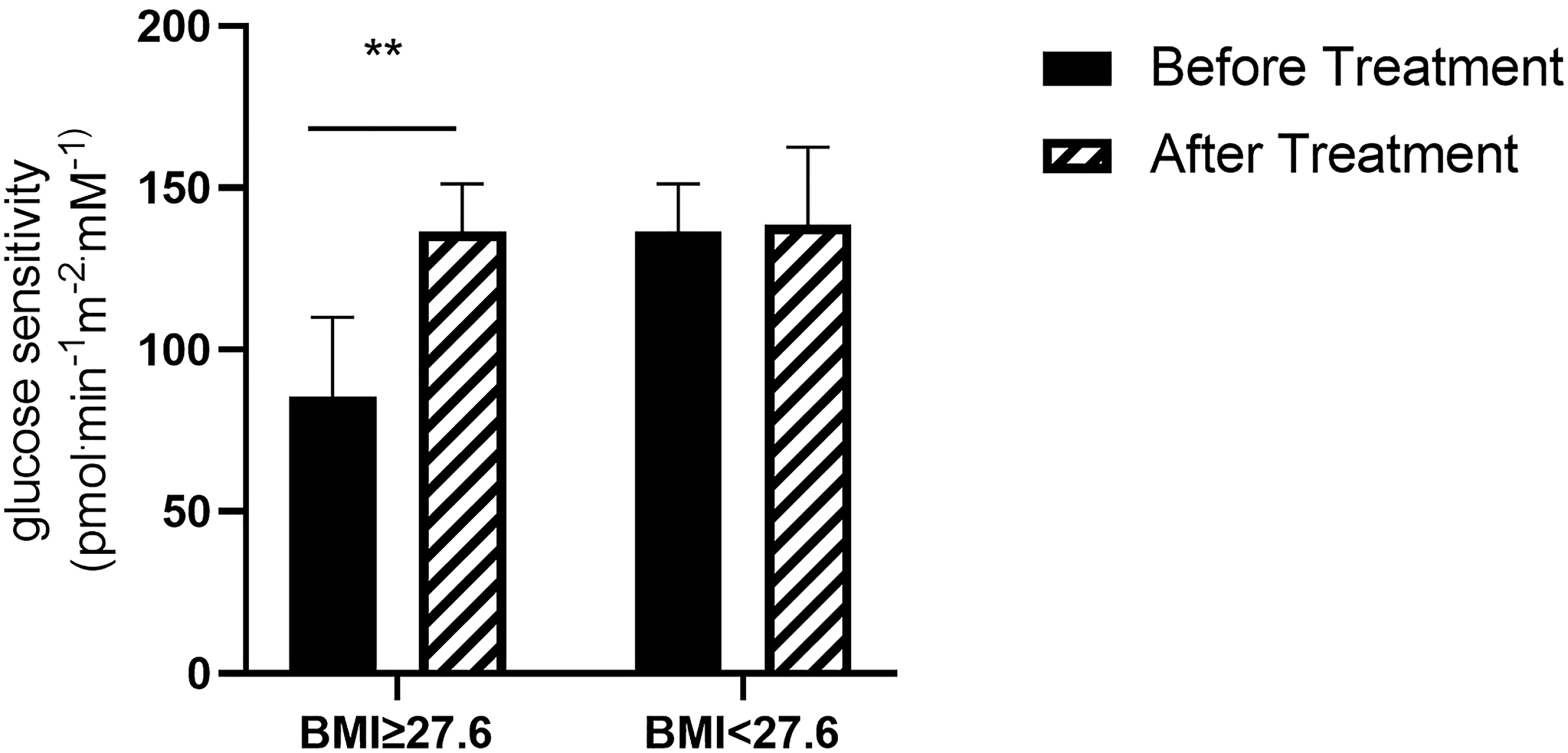
β-cell glucose sensitivity in patients with BMI ≥27.6 kg/m^2^ (N= 7) and BMI < 27.6 kg/m^2^ (N= 8) **p<0.01 before vs after treatment in patients with BMI ≥27.6 kg/m^2^.

We also analyzed whether there was a relationship between change in βCGS and a series of metabolic variables. We found a significant correlation between change in βCGS and BMI (p=0.004). Thus, we compared subjects who had values above and below the median (27.6 kg/m^2^) and found that those with higher BMI had a greater increase in βCGS after therapy with anti-PCSK9 mAb (before: 85.37 ± 24.73; after: 118.62 ± 26.83 pmol min^-1^m^-2^mM^-1^; p=0.007) (**Figure 4**).

**Figure 5:**
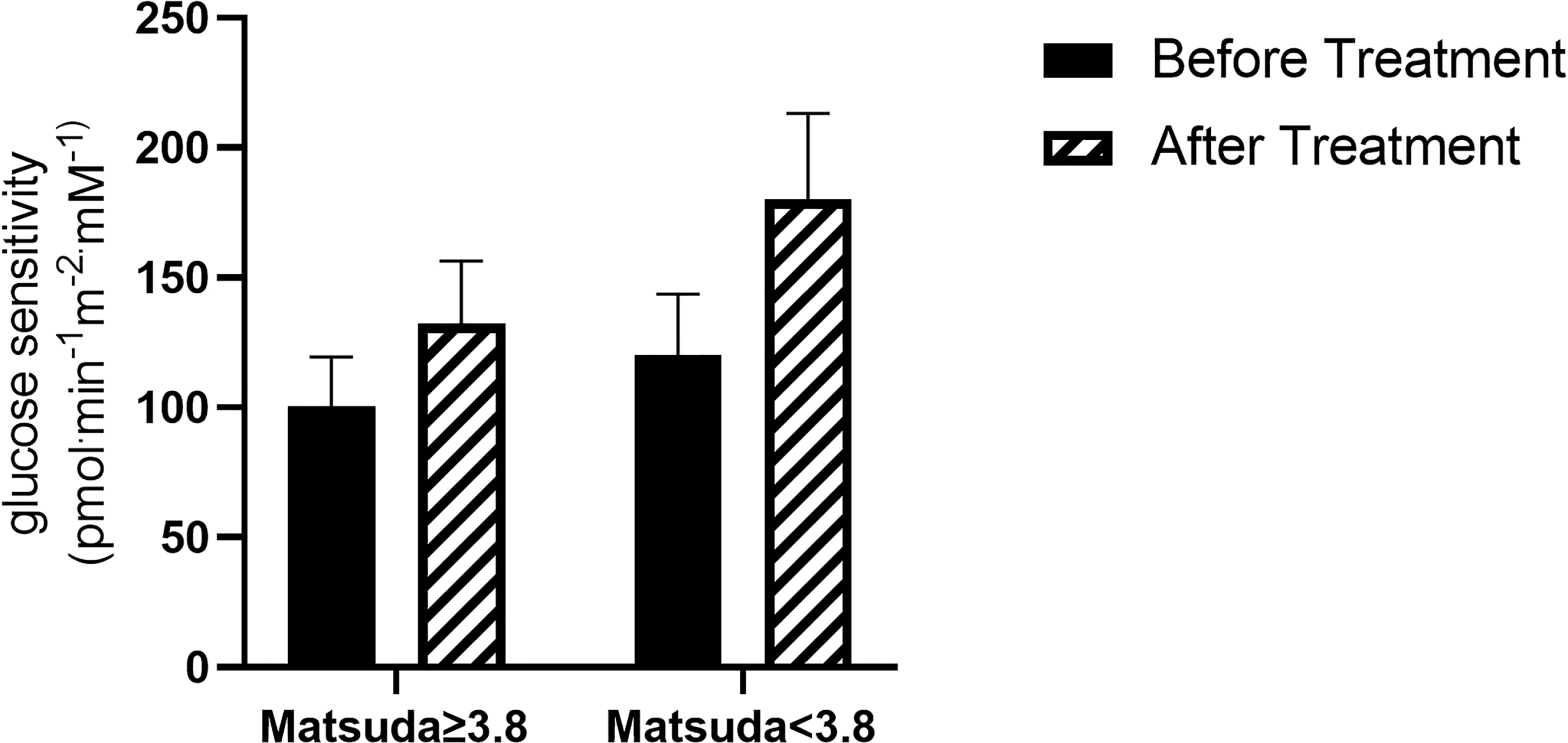
β-cell glucose sensitivity in patients with Matsuda ≥3.8 (N=8) and Matsuda <3.8 (N= 7).

We also found a significant correlation between change in βCGS and Matsuda index (p=0.04), so we analyzed subjects who had values above and below the median (3.8). This subgroup analysis showed a slight, but not statistically significant improvement, in βCGS in the more insulin resistant patients, (before: 131.4 ± 69.8; after: 170.8 ± 92.7 pmol min^-1^m^-2^mM^-1^; p=0.066) (**Figure 5**).

**Figure 6:**
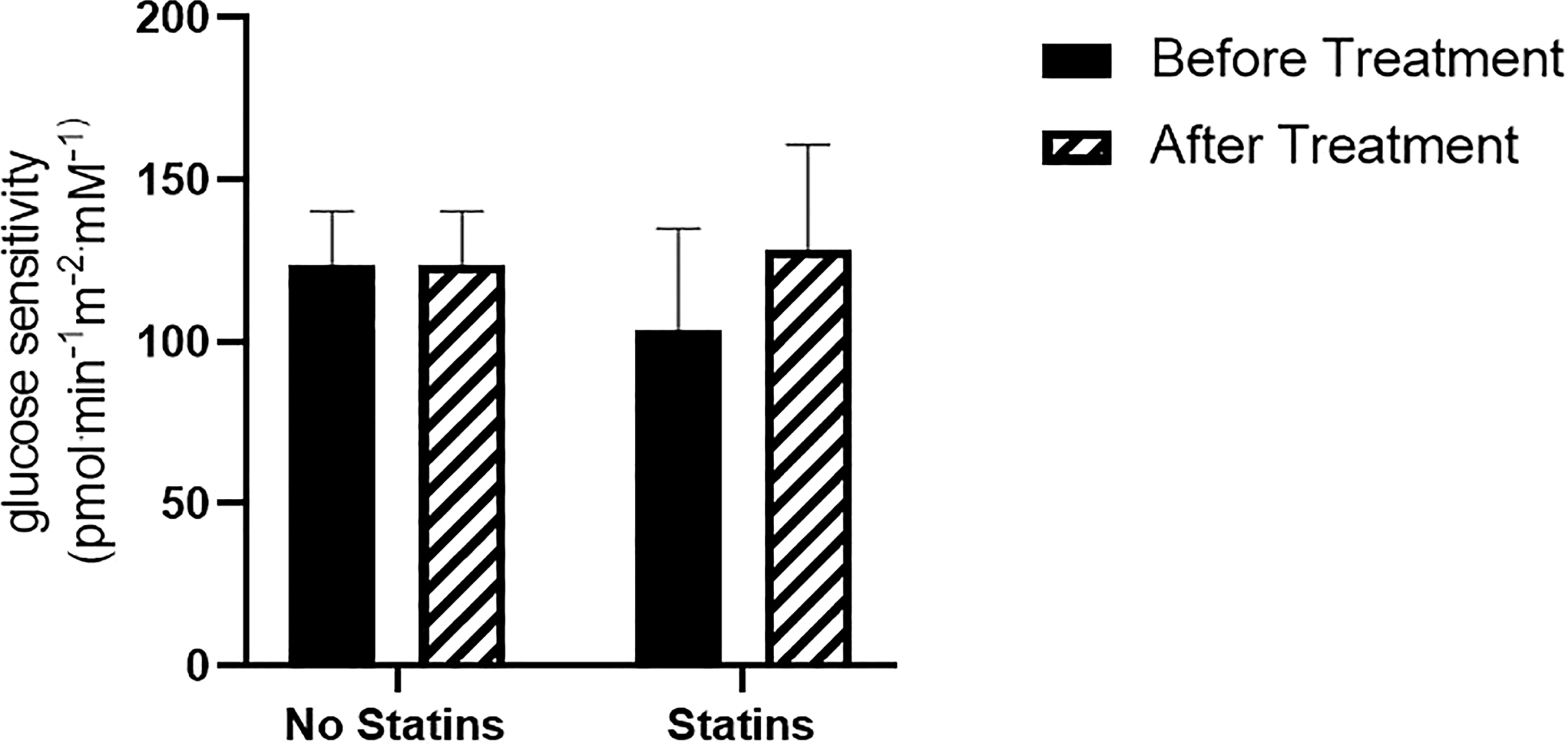
β-cell glucose sensitivity in patients treated with statins (N= 9), and statin intolerant (N= 6).

To exclude a possible interfering effect, we also analyzed the relationship between βCGS change and statin therapy. Even though the analysis showed no significant interaction between these two variables (p=0.96), for exploratory purposes we evaluated changes in βCGS in both treatment groups (Statins/No Statins). We found that there was a greater, though not significant increase in βCGS, in participants on statins (No statins: before: 123.9 ± 14; after: 147.8 ± 23.0 pmol min^-1^m^-2^mM^-1^, p=0.2; Statins: before: 103.8 ± 21.9; after: 128.5 ± 22.7 pmol min^-1^m^-2^mM^-1^, p=0.06) (**Figure 6**).

## Discussion

Our study demonstrates that, after six months of treatment, anti-PCSK9 mAb did not adversely affect glucose tolerance and β-cell function. In contrast, a slight overall improvement in β-cell glucose sensitivity was observed after therapy.

On the other hand, treatment with anti-PCSK9 mAb does not seem to affect insulin sensitivity, as assessed by the Matsuda surrogate insulin sensitivity indexes, suggesting that the slight reduction observed in insulin secretion at 90 min, could be mediated by the improvement in β-cell function.

As explained previously, β-cell glucose sensitivity represents the relationship between insulin secretion and glucose concentration during OGTT, and is a specific and accurate parameter of β-cell secretive function.

To better define the effect of anti-PCSK9 mAb therapy on βCGS, we studied the correlation between βCGS change and other metabolic parameters. We explored whether differences in the BMI, Matsuda index and statin use could predict a different effect of treatment on β-cell function. Our analysis suggests that patients with higher BMI, and those who were more insulin resistant at Matsuda index had greater improvement in β-cell glucose sensitivity after the treatment period compared to those with better metabolic parameters. Moreover, we found a greater, though not significant, increase in βCGS in patients taking statins; a sign that the drug could counteract the negative effect of statins on insulin secretion.

One possible explanation could be that treatment with anti-PCSK9 mAb determines a detectable improvement in hepatic insulin sensitivity only in those with a worse metabolic pattern, higher insulin resistance and BMI.

It is likely that anti-PCSK9 mAb act basically only on the hepatic synthesis of PCSK9. Therefore, there could be an increased expression of PCSK9 in other tissues, including pancreatic islets, which would induce a reduced expression of LDLR in β-cells and therefore a reduced accumulation of intracellular cholesterol, which is known to determine an insulin secretory deficit

The reason why this improvement would be more noticeable in more insulin-resistant subjects and those with higher BMI, could be due to an improvement induced by therapy with anti-PCSK9 mAb. However, the fact that no significant differences in insulin sensitivity (Matsuda) indices were found could be due to the small sample size studied.

Our study design presents several advantages considering that we evaluated both insulin sensitivity and insulin secretion in patients treated with anti-PCSK9 mAb. Firstly, all individuals were evaluated not only through anamnesis and HbA1c but also using the gold standard OGTT, thus allowing us to estimate glucose tolerance and β-cell function. Interestingly, all had comparable glucose, insulin and C-peptide levels at OGTT after treatment. Therefore, OGTT per se is not sufficient to truly identify the metabolic effects of anti-PCSK9 mAb treatment on β-cell function. Only the mathematical modelling of glucose sensitivity allowed us to detect a significant improvement in β-cell function and to distinguish functional changes in a homogenous group of non-diabetic participants undergoing the same treatment. On the other hand, the limits of the study were the small sample size, considering that only 15 subjects were enrolled, the absence of a placebo control group and the fact that there was no blinding.

Despite the small patient cohort, we provide an accurate estimation of the effect of PCSK9 mAb therapy on glucose metabolism and β-cell function. Additional studies with a larger cohort and longer followup are warranted to endorse our findings. This study, however, provides a foundation to plan further studies to identify the beneficial effect of anti-PCSK9 mAb on β-cell function and determine the molecular mechanisms responsible for the dynamic changes that impact β-cells. Moreover, newly released drugs that block PCSK9 synthesis, such as inclisiran, could have a different effect on β-cell function compared to anti-PCSK9 mAb, which only bind extracellular PCSK9 and do not interfere with its intracellular form (24). In contrast, other drugs that do not act on PCSK9, such as bempedoic acid, do not worsen fasting glucose or HbA1c in patients with diabetes or prediabetes, or increase the incidence of new-onset diabetes in patients with normoglycemia compared with placebo (25).

## Conclusions

In conclusion, our study, although it is only an observational pilot study, with a small sample size, no placebo control group and no blinding, demonstrates that treatment with anti-PCSK9 mAb for six months improves β-cell function, confirming, therefore, that therapy with anti-PCSK9 mAb does not lead to alterations in glucose tolerance. This improvement is more evident in subjects who are more insulin-resistant (lower Matsuda) and have higher BMI.

## Data availability statement

The raw data supporting the conclusions of this article will be made available by the authors, without undue reservation.

## Ethics statement

The studies involving human participants were reviewed and approved by Catholic University of Rome Ethics Committee (Prot. 46923/18). The patients/participants provided their written informed consent to participate in this study.

## Author contributions

S.M., generated the data and wrote the manuscript. T. M. generated the data and revised the manuscript, M.P.F contributed to the analysis. G.D.G., CMA.C and F.C. contributed to the enrollment of patients. F.I., U.C. and G.C. revised the manuscript. A.M. and A.P. contributed to the analysis and revised the manuscript. A.G. reviewed/edited manuscript.
